# Morphological and Molecular Characterization of *Phasmarhabditis huizhouensis* sp. nov. (Nematoda: Rhabditidae), a New Rhabditid Nematode from South China

**DOI:** 10.1371/journal.pone.0144386

**Published:** 2015-12-16

**Authors:** Ren-E Huang, Weimin Ye, Xiaoliang Ren, Zhongying Zhao

**Affiliations:** 1 School of Life Sciences, Tsinghua University, Beijing, China; 2 Department of Biology, Faculty of Science, Hong Kong Baptist University, Hong Kong, China; 3 Nematode Assay Section, Agronomic Division, North Carolina Department of Agriculture & Consumer Services, Raleigh, North Carolina, United States of America; Australian Museum, AUSTRALIA

## Abstract

The genus *Phasmarhabditis* is an economically important group of rhabditid nematodes, to which the well-known slug-parasite *P*. *hermaphrodita* belongs. Despite the commercial use of *Phasmarhabditis* species as an attractive and promising approach for pest control, the taxonomy and systematics of this group of rhabditids are poorly understood, largely because of the lack of diagnostic morphological features and DNA sequences for distinguishing species or inferring phylogenetic relationship. During a nematode sampling effort for identifying free-living relatives of *Caenorhabditis elegans* in Huizhou City, Guangdong, China, a novel species belonging to the genus *Phasmarhabditis* was isolated from rotting leaves. Detailed morphology of the gonochoristic *P*. *huizhouensis* sp. nov. was described and illustrated. The adult female has a robust body, a relatively short and wide buccal capsule conjoined by a rhabditiform pharynx. Females are characterized by a short cupola-shaped tail end bearing a slender pointed tip, with the junction flanked by a pair of ‘rod-like’ phasmids. Males have an open peloderan bursa that is supported by 9 pairs of genital papillae and 1 terminal pair of phasmids. *P*. *huizhouensis* sp. nov. is morphologically very similar to the type species *Phasmarhabditis papillosa* but is distinguishable by its male caudal traits. The new species is readily differentiated from other taxa in the genus by its female tail shape. Molecular phylogenetic inferences based on small subunit (SSU) and the D2-D3 domain of large subunit (LSU) ribosomal DNA genes reveal that *P*. *huizhouensis* sp. nov. forms a unique branch in both phylogenies which is genetically related to *P*. *hermaphrodita* and other parasites such as *Angiostoma* spp. The host associations of *P*. *huizhouensis* sp. nov. and its ability to parasitize slugs are unknown.

## Introduction

During our sampling of free-living nematodes in south China’s Guangdong Province, a novel rhabditid nematode was recovered and reared on standard *C*. *elegans* culture media seeded with *Escherichia coli* strain OP50. The rhabditid has a relatively short and wide buccal capsule with simple metastegostom ridges, female ‘rod-like’ prominent phasmids and male open peloderan bursa with 10 pair of bursal papillae, morphologically belonging to the ‘*Papillosa*’ group in the genus *Pellioditis* Dougherty, 1953 *sensu* Sudhaus [1–3]. However, the revision of *Pellioditis* by Andrássy was disparate, with the generic name assigned to the revised ‘*Papillosa*’ group being completely different to that assigned by Sudhaus. Andrássy established a new genus of *Phasmarhabditis* including *Pelodera papillosa* (syn. *Pellioditis papillosa*) as the type species, *P*. *hermaphrodita* and *P*. *neopapillosa* from the ‘*Papillosa*’ group, and two other species: *Rhabditis* (*Cephaloboides*) *nidrosiensis* with a marine habitat and *R*. (*C*.) *valida* with a littoral habitat [4–5]. Nevertheless, in a recent revision to the family Rhabditidae by Sudhaus, both species of *nidrosiensis* and *valida* were classified into another genus (*Buetschlinema* Sudhaus, 2011) and the remaining *Phasmarhabditis* taxa were replaced in *Pellioditis* [3]. In Sudhaus’ revision of the original *Pellioditis*, all species were moved into other genera except the ‘*Papillosa*’ group [3], whereas only this group was transferred in the revision by Andrássy [5]. In other words, the revised *Pellioditis sensu* Sudhaus [1–3] is a taxon basically equivalent to *Phasmarhabditis sensu* Andrássy [4–5] containing stem species of the ‘*Papillosa*’ group, i.e., *P*. *papillosa*, *P*. *phasmarhabditis* and *P*. *neopapillosa*. Though the taxon *Pelodytes* Schneider, 1895 with type species *hermaphrodites* (= *hermaphrodita*) preceded both taxa of *Pellioditis* and *Phasmarhabditis*, it was preoccupied by an amphibian genus [6–8]. The nomenclature ‘*Pellioditis*’ has priority over ‘*Phasmarhabditis*’, however, both generic names assigned to a synonymous group of rhabditids were simply indicative of diverse perspectives of revisions by Sudhaus and Andrássy respectively. To avoid taxonomic confusion, the new species is herein named as *Phasmarhabditis huizhouensis* sp. nov., with its specific epithet derived from its locality (Huizhou City).

*Phasmarhabditis hermaphrodita* is a well-known deadly parasite of slug pests of agricultural and horticultural crops. It is known to be capable of killing many species of slugs from several families. This nematode has been studied intensively and formulated into an effective biological control agent called Nemaslug® which was sold in Europe [9–13]. Recently, *P*. *phasmarhabditis* was reported for the first time in North America from the invasive slugs *Deroceras reticulatum*, *D*. *laeve* and *Lehmannia valentiana* [8]. *P*. *neopapillosa* is a close relative of *P*. *hermaphrodita* that is morphologically indistinguishable by females or hermaphrodites. The species can be differentiated by the frequency of males. Males are common in *P*. *neopapillosa*, but rare or absent in *P*. *hermaphrodita* [14]. Although *P*. *papillosa* was isolated from decaying organic materials, this species is also associated with terrestrial snails and slugs [15–16]. Later, *P*. *tawfiki* was described from Greater Cairo, Egypt, where it was isolated from the snail *Eobania vermiculata* Müller and the slug *Limax flavus* Linneaus, with males rare [17]. In addition, two undescribed species were found associated with the slug *D*. *reticulatum* and its relative *Ariostralis nebulosa* in South Africa [18], and another unidentified isolate was reported to infect the earthworm *Lumbricus terrestris* in Champaign, Illinois, USA [19]. All these *Phasmarhabditis* species have similar host associations. They are either slug-parasites or associated with slugs, snails or earthworms. Recent molecular studies revealed that all *Phasmarhabditis* species are in a monophyletic group [8].

## Materials and Methods

### Nematode isolation, culture and microscopic observation

*Phasmarhabditis huizhouensis* sp. nov. was described and illustrated using a wild isolate ZZY0412 that was recovered from rotting leaves in Kowloon Peak of Huizhou City, Guangdong Province, China (our sampling activity was conducted using rotting plant leaves, so no specific permissions were required, and the field studies did not involve endangered or protected species). Samples were placed onto a clean agar plate. Nematode individuals were picked out then cultured at room temperature using the *C*. *elegans* food (*E*. *coli* OP50). Measurements [20], drawings and descriptions were conducted from quiescent adult worms incubated at 4°C for 12h after recovery from 3-day culture plates. Differential interference contrast (DIC) images were taken with an Eclipse Ti Inverted Microscope (Nikon). Scanning electron microscopy (SEM) of *P*. *huizhouensis* sp. nov. was performed either using the Hitachi_S-450 scanning electron microscope at Hong Kong Baptist University or the Hitachi_S-3400N at China Agricultural University by the methods described previously [21]. Permanent slides were made using the specimens fixed in 3% formaldehyde, which were subsequently mounted in anhydrous glycerin under a paraffin seal after gradual dehydration of nematodes on a hot plate at 70°C[22].

### DNA sequencing and phylogenetic analysis

For molecular analysis, genomic template DNA of *Phasmarhabditis huizhouensis* sp. nov. strain ZZY0412 was extracted using PureLink^TM^ Genomic DNA Mini Kit (Invitrogen, USA). SSU rDNA gene fragment of 1633 base pairs (bp) was amplified by PCR using three sets of universal primers shown below: (1) SSU18A (5’-AAAGATTAAGCCATGCATG-3’) and SSU26R (5’-CATTCTTGGCAAATGCTTTCG-3’) [23–24]; (2) RHAB1350F (5’-TACAATGGAAGGCAGCAGGC-3’) and RHAB1868R (5’-CCTCTGACTTTCGTTCTTGATTAA-3’) [23, 25]; and (3): SSU24F (5’-AGRGGTGAAATYCGTGGACC-3’) and SSU18P (5’-TGATCCWMCRGCAGGTTCAC-3’) [26–27]. Forward and reverse primer sequences for amplification of 902-bp D2-D3 LSU rDNA sequence were No. 391 (5’-AGCGGAGGAAAAGAAACTAA-3’) [28–29] and No. 501 (5’-TCGGAAGGAACCAGCTACTA-3’) [28, 30], respectively. PCR amplification and sequencing were performed as previously described [21]. The SSU and the D2-D3 domain of LSU rDNA sequences were deposited in GenBank database with accession numbers of KP017252 and KP017253 respectively, and were aligned against the sequences from GenBank to identify closely related species.

The model of base substitution was evaluated using MODELTEST [31–32]. The estimates of base frequencies, the proportion of invariable sites, the gamma distribution shape parameters and substitution rates suggested by the Akaike Information Criterion were used in phylogenetic analyses. Bayesian analysis was performed to confirm the tree topology for each gene separately using MrBayes 3.1.0 [32] running the chain for 1 × 10^7^ generations and setting the “burnin” at 2,5000 which is 25% of the run. The sampling and printing frequencies are 100 and 1000 respectively. We used the Markov Chain Monte Carlo (MCMC) method within a Bayesian framework to estimate the posterior probabilities of the phylogenetic trees using 50% majority rule [33]. Tracer was used for assessing and summarizing the posterior estimates of the various parameters sampled by the Markov Chain and confirming that the burnin was approaching stationarity (http://tree.bio.ed.ac.uk/software/tracer).

### Nomenclatural acts

The electronic edition of this article conforms to the requirements of the amended International Code of Zoological Nomenclature, and hence the new names contained herein are available under that Code from the electronic edition of this article. This published work and the nomenclatural acts it contains have been registered in ZooBank, the online registration system for the ICZN. The ZooBank LSIDs (Life Science Identifiers) can be resolved and the associated information can be viewed through any standard web browser by appending the LSID to the prefix "http://zoobank.org/". The LSID for this publication is: urn:lsid:zoobank.org:pub:47BE7062-4ECC-41B3-85CE-C3A53BE6C399. The electronic edition of this work was published in a journal with an ISSN, and has been archived and is available from the following digital repositories: PubMed Central, LOCKSS.

## Results

### *Phasmarhabditis huizhouensis* Huang, Ye, Ren & Zhao sp. nov.

**Fig 1:**
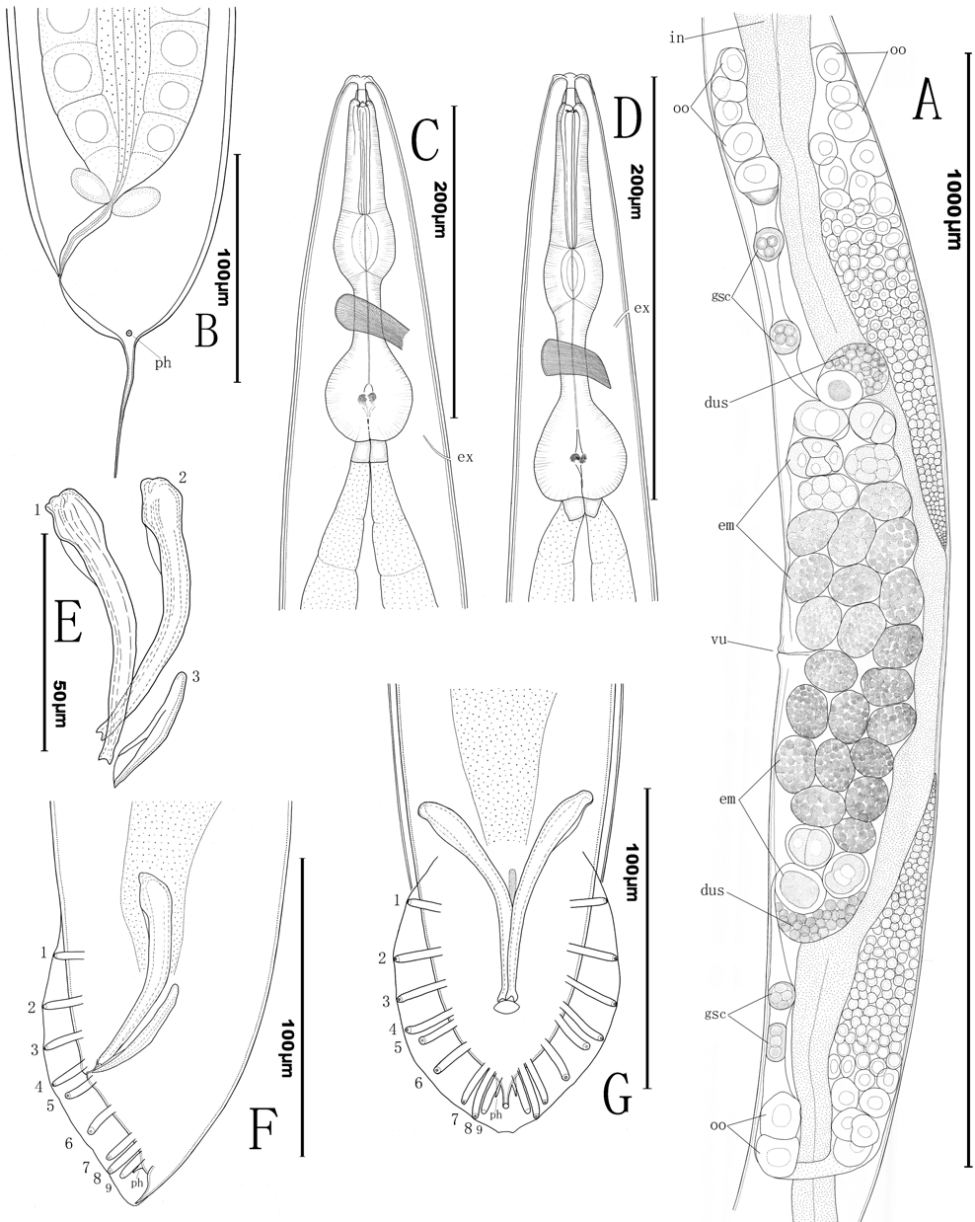
Line drawings of *Phasmarhabditis huizhouensis* sp. nov. (A) Female reproductive system (in: intestine; oo: oocytes; gsc: aggregated sperm cells; dus: distal part of uteri filled with sperms; em: embryos; vu: vulva). (B) Female tail (ph: phasmid). (C) Anterior region of female (ex: excretory pore). (D) Anterior region of male. (E) Spicules and gubernaculum (1: spicule in dorsal view; 2: spicule in ventral view; 3: gubernaculum from sublateral view). (F) Lateral view of male tail (1–9: nine pairs of genital papillae; ph: phasmid). (G) Ventral view of male tail with an open peloderan bursa supported by 9 pairs of genital papillae and 1 terminal pair of phasmids.

**Fig 2:**
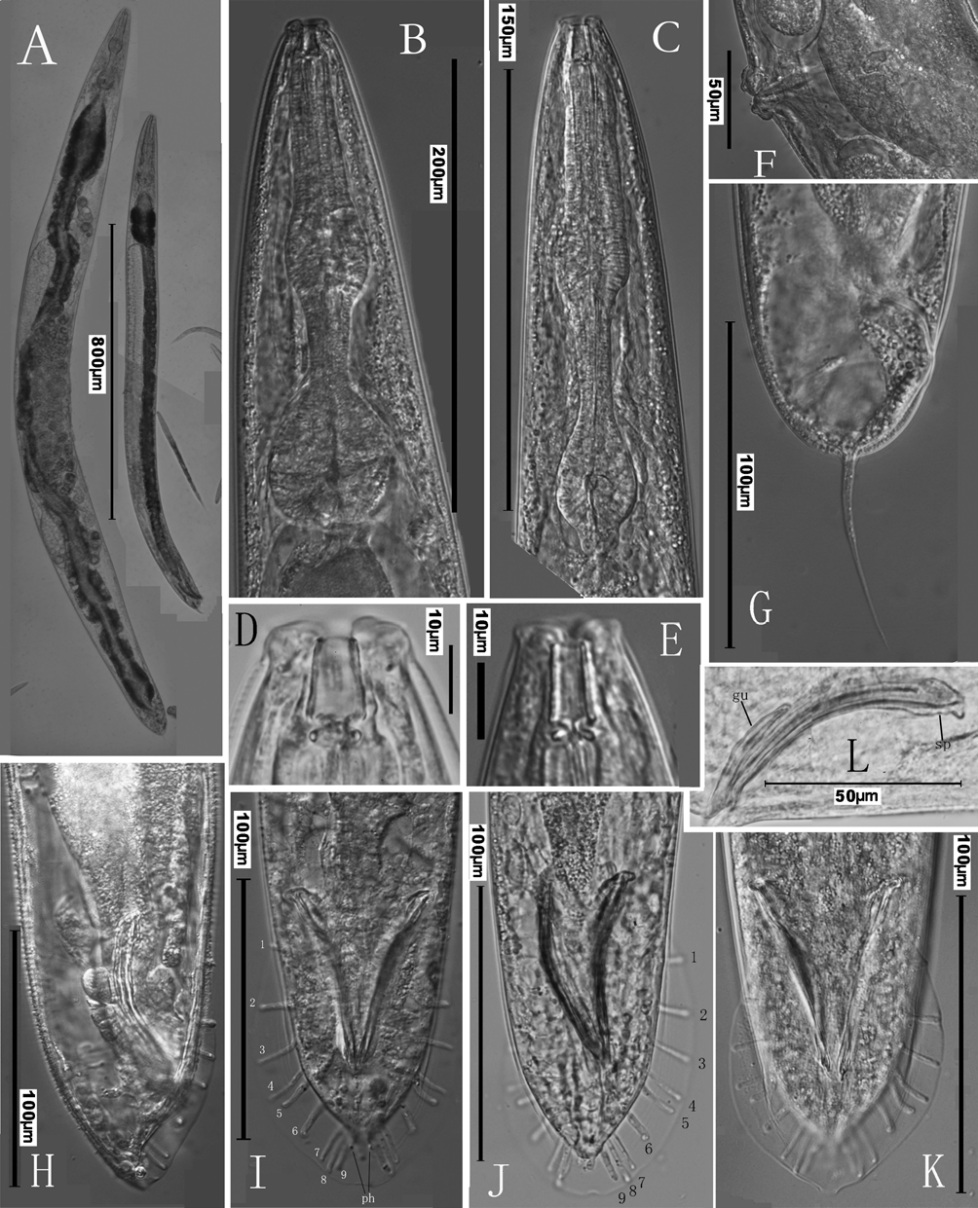
Micrographs of *Phasmarhabditis huizhouensis* sp. nov. (A) Overview of female (left) and male (right) adults. (B) Pharyngeal region of an adult female. (C) Pharyngeal region of an adult male. (D) Anterior end (stoma) of a female. (E) Anterior end (stoma) of a male. (F) Vulva. (G) Female tail. (H) Lateral view of male tail. (I) and (J) Ventral view of male tails showing ten pairs of bursal papillae [nine pairs of genital papillae (1–9) and one terminal pair of phasmids (ph) (I)]. (K) Ventral view of the male caudal bursa. (L) Spicules (sp) and gubernaculum (gu).

**Fig 3:**
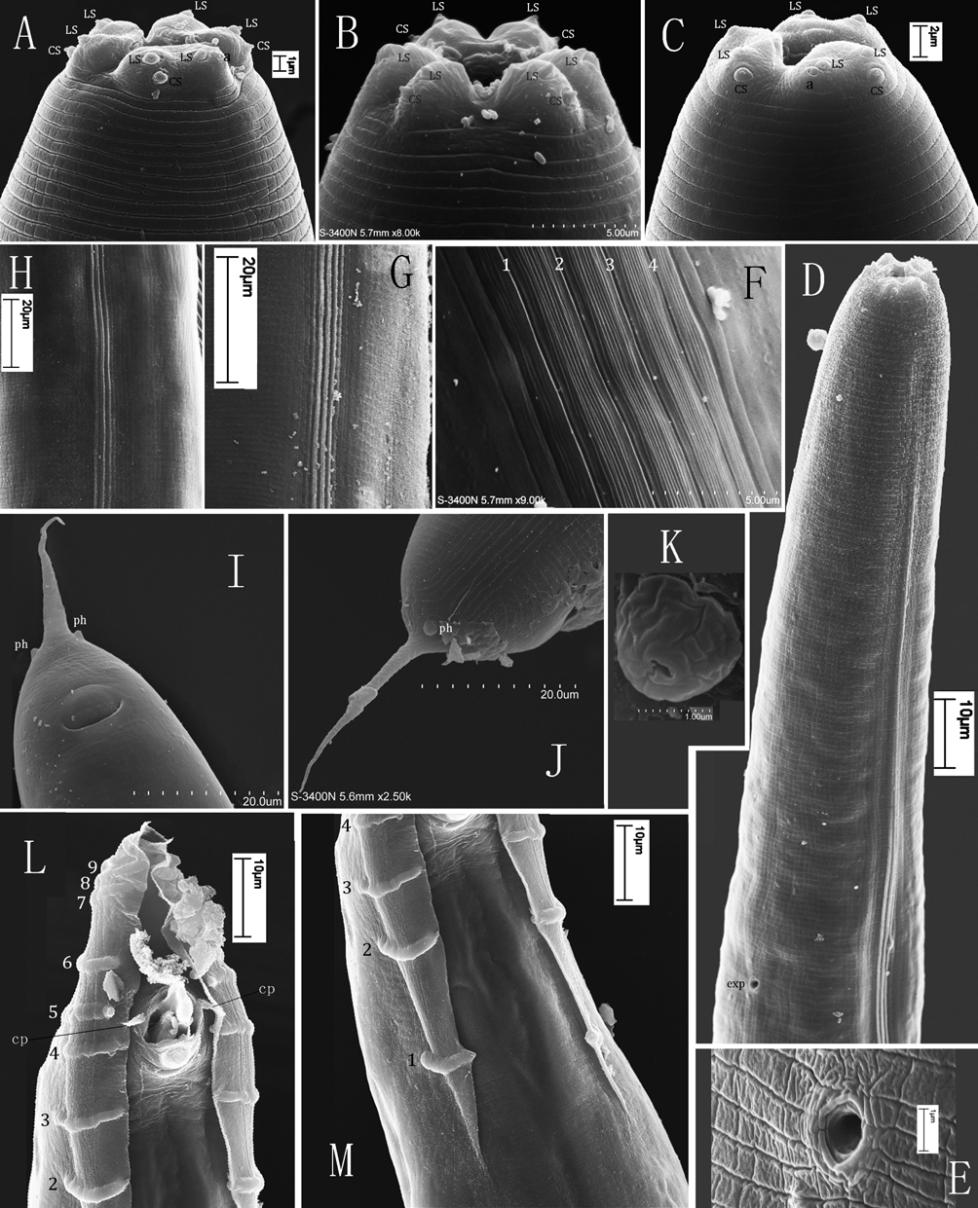
Scanning electron micrographs of *Phasmarhabditis huizhouensis* sp. nov. (A) and (B) Head of females (LS: labial sensilla; a: amphid; CS: cephalic sensilla). (C) Head of male. (D) Anterior region of male showing excretory pore (exp). (E) Magnification of the excretory pore. (F) and (G) Lateral fields of females (1–4: 4 incisures compose the three middle ridges/bands). (H) Lateral field of male. (I) and (J) Ventral and lateral views of female tails (ph: phasmid). (K) Magnification of female phasmid. (L) and (M) Male caudal region with an open bursa as well as its 9 pairs of genital papillae (1–9) (cp: cloacal papillae). [(B), (F), (I), (J) and (K) were captured by the Hitachi_S-3400N scanning electron microscope, others by the Hitachi_S-450].

urn:lsid:zoobank.org:act:B24611B5-9A23-4B2D-BAC4-D6EA2647E57B (Figs 1–3)

### Description

#### Adults

Cuticle surface smooth or finely annulated. Lateral field longitudinally striated, often bearing 7–11 lines (ridges or incisures) with 3 prominent ridges or bands in the middle (Fig 3F–3H). Lip region flattened anteriorly, slightly higher in females than males. Six lips bearing two circles of sensilla arranged in three pairs: one dorsal and the others sub-ventral, encircling a triangular mouth opening (Fig 3A–3C). Each lip with one apically-inserted, papilliform sensillum. Lateral lips present a pair of small, pore-like amphidial openings, located near the labial sensilla. The other four sub-lateral lips each with 1 papilliform cephalic sensillum posterior to the labial one (Fig 3A–3C). Stoma capsule relatively wide and short (15–24 μm × 5–8 μm), isomorphic/isotropic. Anterior part of the stoma (Cheilostom) cuticularized; gymnostom together with prostegostom and mesostegostom forming main part of the stoma, cylinder-like and slightly funnel-shaped; metastegostom ridges present, but lacking teeth or denticles; telostegostom short (Fig 2D and 2E). Anterior part of pharynx (= pro- + meta-corpus) slightly longer than posterior (= isthmus + basal bulb). Metacorpus muscular, forming swollen median bulb (25–52 μm in diam.). Isthmus distinct. Nerve-ring encircling posterior region of isthmus. Basal bulb pyriform (39–71 μm in diam.) with valvular apparatus, but posterior structure of haustrulum obscure in many specimens. Pharyngo-intestinal valves (cardia) present. Excretory pore observed behind the base of basal bulb by light microscopy in a few females, but only *ca* 106 μm from anterior end revealed by SEM examination for one male (Fig 3D). Measurements are listed in Table 1.

**Table 1:** Morphometrics of *Phasmarhabditis huizhouensis* sp. nov. [20]. All measurements are in μm and shown in the form: mean ± s.d. (range).

Character	Females	Males
	(n = 16)	(n = 14)
L	1875.19 ± 340.84 (1333.07–2341.16)	1282.11±247.97 (907.89–1669.21)
a	13.97 ± 0.94 (12.61–15.97)	14.69±1.44 (12.11–17.3)
b	8.33±1.05 (6.39–10.47)	6.6±0.79 (5.3–7.76)
c	20.55±3.3 (15.5–26.86)	26.83±3.54 (20.85–34.27)
c’	1.61±0.17 (1.28–2.02)	1.14±0.18 (0.90–1.47)
V	52.41±0.85 (50.78–54.01)	–
Stoma length	19.68±1.98 (16.84–23.49)	17.8±1.78 (15.1–20.47)
Stoma diam. ^1^	6.53±0.47 (5.91–7.89)	5.66±0.48 (5.03–6.49)
Pharynx length	214.59±19.62 (183.93–251.02)	189.04±12.01 (168.84–211.45)
Anterior part of pharynx length	117.17±10.34 (101.03–138.93)	104.54±5.91 (91.5–112.1)
Posterior part of pharynx length	99.07±10.2 (84.72–117.17)	85.73±7.3 (78.39–103.03)
Distance from anterior end to	220.2±19.98 (188.55–255.89)	195.43±12.71 (176.88–222.22)
the base of pharynx		
Diam. of median bulb	37.85±7.11 (24.16–51.68)	28.94±2.98 (25.17–34.23)
Diam. of terminal bulb	53.24±9.64 (39.06–71.14)	40.91±6.41 (27.85–52.68)
Body diam.	134.38 ± 23.95 (85.5–171)	88.09±15.45 (65.14–113.99
Vulva body diam.	133.45 ± 20.98 (85.5–162.85)	–
Vulva to anus distance	800.22±169.49 (518.22–1053.37)	–
Anal body diam.	57.22 ± 5.89 (42.62–67.11)	41.02±6.99 (30.87–55.17)
Tail length	91.14±6.46 (80.81–105.58)	47.28±8.06 (35.54–61.41)
Length of female pointed tip	56.03±4.99 (48.32–63.97)	–
Spicule length	–	69.96±5.62 (61.07–81.88)
Gubernaculum length	–	35.44±3.07 (29.9–41)
Gubernaculum length as % spicule length	–	50.4±2.03 (48–55)

^1^ Measured in the middle of the stoma; diam. denotes diameter.

#### Female

Body robust, tapering gradually to a blunt anterior end. Gonad didelphic, amphidelphic; anterior branch on the right of intestine, but posterior arm to the left. Vulva located near the middle of entire body (V = 51–54) with lips protruding (Fig 2F). Paired ovaries reflexed opposite to the vulva. Germ cells in both distal parts of ovaries arranged in a central rachis with proximal cells in elongation. Maturing oocytes proximally located in a single row at reflexed regions of both ovaries. Oviduct relatively long (127–221 μm). One or more aggregations of sperm cells visible occasionally but small (24–53 μm × 20–43 μm), each containing two or more sperm cells. Uteri often with numerous embryos irregularly arranged. Many embryos usually carrying unhatched larvae. Both distal parts of uteri (20–69 μm × 36–73 μm) filled with sperm cells, sometimes visible. Tail short and cupola-shaped with a slender pointed tip in the middle (Fig 2G). The pointed tip varying in length from 48 to 64 μm. Phasmids ‘rod-like’ papilliform in SEM (1.6–2.2 μm in diam.), flanking the junction between the cupola-shaped tail end and its slender tip (Fig 3I–3K).

#### Male

Males of *Phasmarhabditis huizhouensis* sp. nov. are common in culture. Testis single-armed, located on right of intestine with anterior end reflexed ventrally. The reflexed part approximately extending for 300–500 μm. Spermatogonia arranged in two to four rows; maturing spermatocytes situated proximally in two to three rows; then spermatids in multiple rows present in remainder of testis. Caudal region enveloped by an open medium-developed peloderan (tail-encompassing) bursa (88–95 μm × 69–78 μm) (Fig 3L and 3M). 10 pairs of bursal papillae present, comprising 9 pairs of genital papillae (GP) or rays (1+1+1+2+1+3) and 1 terminal pair of papilliform phasmids (ph) (Fig 2I). GP1, GP2, and GP3 precloacally standing; GP4 and GP5 cloacally situated; the rest post-clocacally located. GP1, GP2, GP3, GP4, and GP8 extending to the velum edge or near it. The sensory tip of GP5 lying to the dorsal surface; GP9 also appears dorsal (Fig 3L). An additional pair of small cone-shaped papillae flanking the cloaca (Fig 3L). Spicules 61–82 μm long, paired, separated, but in “Y” shape from ventral view when jointed by gubernaculum. Each spicule shaft slightly expanded in anterior end then gradually tapered to a distal small forked terminus (an obtuse tip divided by shallow indentation) (Fig 2L). Gubernaculum short, only half spicule length. Phasmids in fine cone shape, located posterior to GP9 where flanking the tail terminal tip (Fig 2I). Tail short conoid, tapered to a pointed terminus.

### Type locality

The type specimens of *Phasmarhabditis huizhouensis* sp. nov. were derived from Strain ZZY0412, which was established from individuals isolated from rotting leaves in Kowloon Peak of Huidong County, Huizhou City, Guangdong Province, P.R. China (22°59′N, 114°43′E) by rearing on NGM agar plate seeded with colibacillus *Escherichia coli* strain OP50.

### Type materials

Fifteen permanent slides containing paratype males and females of *Phasmarhabditis huizhouensis* sp. nov. strain ZZY0412 were deposited in the United States Department of Agriculture Nematode Collection (USDANC), Beltsville, MD, USA (T-6557p to T-6571p). Additional specimens fixed in 3% formalin were deposited in the laboratory of the Department of Biology, Hong Kong Baptist University, Hong Kong, China.

Living worms of the type isolate ZZY0412 were cryogenically preserved and deposited in the Department of Biology, Hong Kong Baptist University, Hong Kong, China.

### Diagnosis and relationships

*Phasmarhabditis huizhouensis* sp. nov. is characterized by the following features, including gonochoristic reproductive mode, large female body size (L = 1.3–2.3 mm; ‘a’ value = 12.6–16), longitudinally striated lateral field with 3 distinct ridges/bands, relatively short and wide buccal capsule with simple metastegostom ridges, and a rhabditiform pharynx. Female didelphic-amphidelphic reproductive system carrying numerous embryos, vulva mid-body located (V = 51–54); a short cupola-shaped tail end conjoining a slender pointed tip which is flanked by a pair of ‘rod-like’ phasmids. Male open peloderan bursa medium-developed that is supported by 9 pairs of genital papillae and 1 terminal pair of phasmids; spicules paired, with small forklike distal termini (obtuse tips divided by shallow indentation) and a short gubernaculum.

*Phasmarhabditis huizhouensis* sp. nov. morphologically belongs to the genus because of its large female body size, relatively short and wide buccal capsule, longitudinally striated lateral field, female ‘rod-like’ phasmids, and male open peloderan bursa as well as its 10 pairs of bursal papillae. Within the genus *Phasmarhabditis*, *P*. *huizhouensis* sp. nov. is clearly distinguished from *P*. *hermaphrodita*, *P*. *neopapillosa* and *P*. *tawfiki* by the female/hermaphroditic tail shape (a short cupola joining a slender pointed tip vs. a relatively short conoid gradually tapered to a pointed terminus) [14, 17]. *P*. *huizhouensis* sp. nov. shares natural habitats (e.g. isolated from decaying organic substances) and the female tail shape (cupola-shaped with a slender tip) with *P*. *papillosa*, but the new species can be differentiated from *P*. *papillosa* by a significantly longer spicule length (61–82 μm vs. *ca* 30 μm for *P*. *papillosa*, as measured from Fig 1B [15]) and a less-developed peloderan bursa (88–95 μm × 69–78 μm vs. 141 μm × 107 μm—the size for *P*. *papillosa* in Fig 1B [15]). In addition to these *Phasmarhabditis* species, there are remaining taxa of *P*. *incilaria*, *P*. *mairei* and *P*. *pellio* in the genus *Pellioditis sensu* Sudhaus [3]. *P*. *huizhouensis* sp. nov. is different from *P*. *pellio* in female body size: L = 1.3–2.3 mm vs. 0.79–0.90 mm, and male spicule length: 61–82 μm vs. only 21–26 μm for *P*. *pellio* [34]. The new species differs from *P*. *incilaria* in the stoma morphology (without ornamentation on metastegostom ridges vs. bearing bristle-like denticles) [5]. The description of *P*. *mairei* is unavailable and this species was considered to be ‘species inquirenda’ by Andrássy [5]. Within the genus *Pellioditis sensu* Andrássy [5], *P*. *huizhouensis* sp. nov. is distinguished from all species in the genus by the typology of stoma (relatively short and wide without ornamentation on glottoid apparatus vs. at least four times as long as its width with warts on metastegostom ridges).

### Molecular analysis

The best scores from the query of 1633-bp SSU gene fragment of *P*. *huizhouensis* sp. nov. against the GenBank database are the sequences from 3 isolates of unidentified *Phasmarhabditis* species, 6 isolates of *P*. *hermaphrodita* and 1 isolate of *P*. *neopapillosa*. SSU sequence alignments between *P*. *huizhouensis* sp. nov. and the most similar isolates revealed that the minimal sequence divergence is from an unidentified species ITD046 from North America (KM510210) [8], with 2.88% divergence over complete coverage. The four US *P*. *hermaphrodita* isolates of ITD290 (KM510209), ITD272 (KM510208), ITD207 (KM510207), and ITD056 (KM510206) [8] have a uniform divergence of 3.55% over complete coverage. The sequence of *P*. *neopapillosa* (FJ516754) gives 95% coverage yielding 2.94% divergence. The best match to the 902-bp D2-D3 sequence of *P*. *huizhouensis* sp. nov. from GenBank is another unidentified *Phasmarhabditis* species EM434 (EU195967), with 5.78% divergence over 95% coverage. D2-D3 sequence alignments between *P*. *huizhouensis* sp. nov. and the four US *P*. *hermaphrodita* isolates of ITD290 (KM510296), ITD272 (KM510195), ITD207 (KM510194), and ITD056 (KM510193) [8], yielded only 59% coverage with a uniform divergence of 7.84%. Comparisons of the sequences of the SSU and D2-D3 domain of the LSU rDNA genes between *P*. *huizhouensis* sp. nov. and its relatives further support that our rhabditid nematode is a novel species.

**Fig 4:**
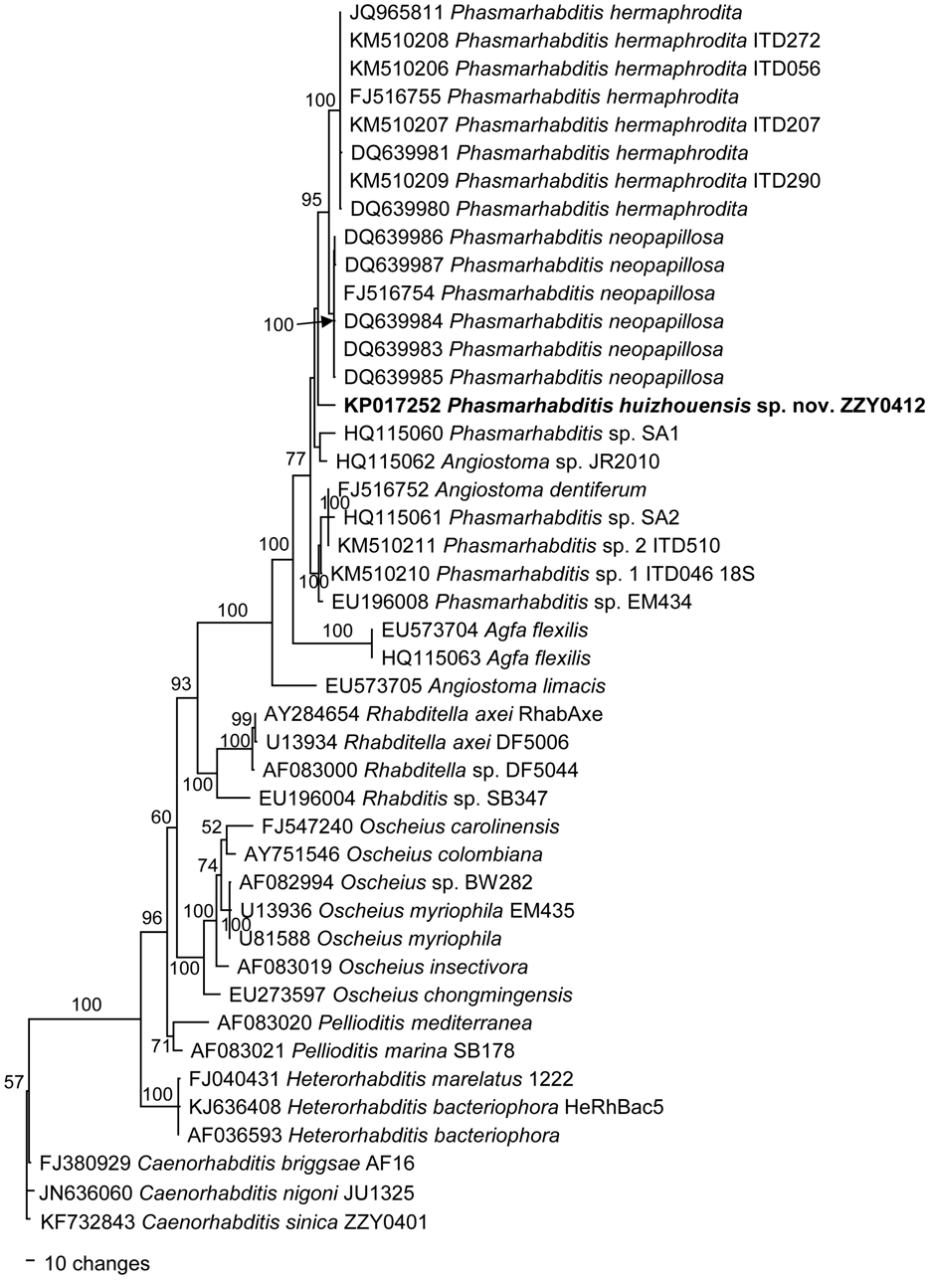
Bayesian tree inferred from SSU rDNA sequences. Posterior probability values exceeding 50% are given on appropriate clades. GenBank and strain ID associated with DNA sequences are shown on the left and right of the species name respectively.

**Fig 5:**
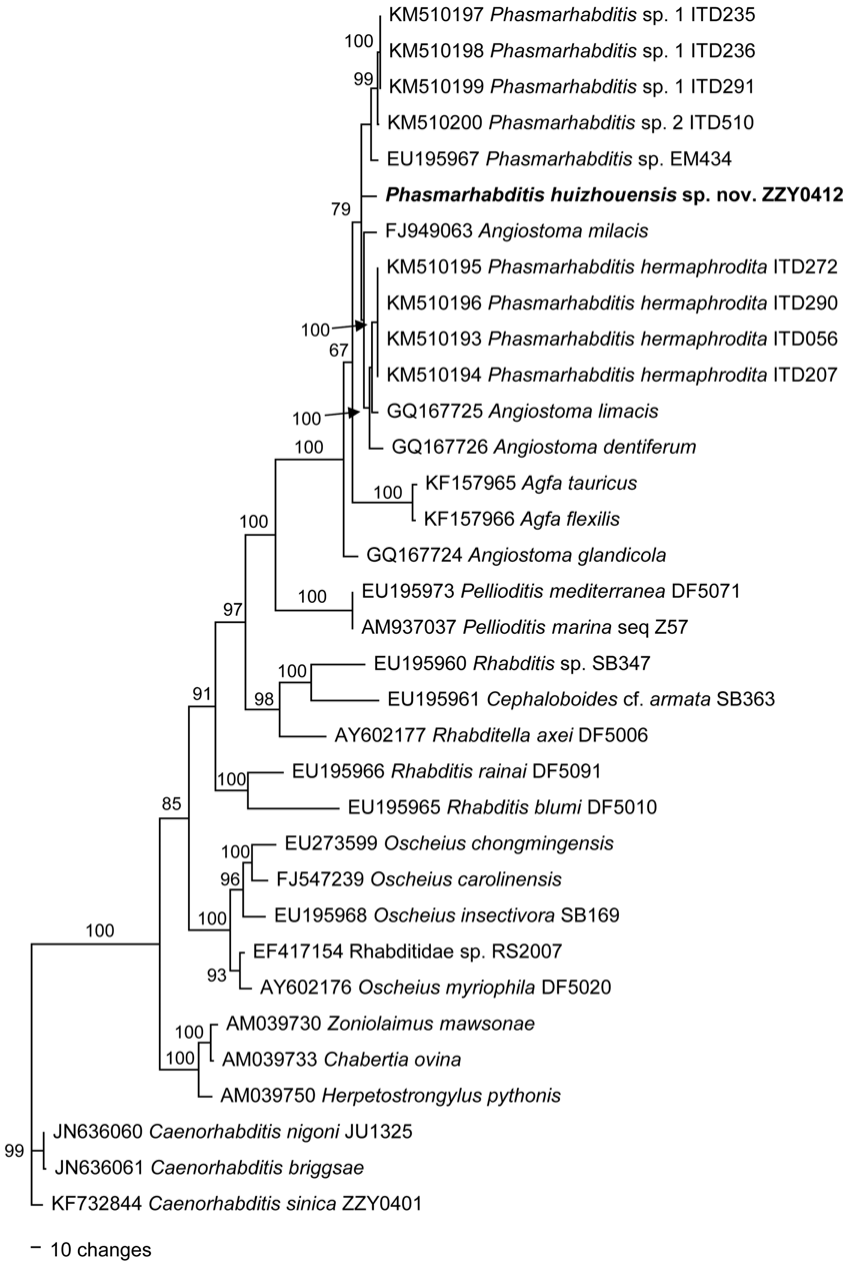
Bayesian tree inferred from LSU D2-D3 rDNA sequences. Posterior probability values exceeding 50% are given on appropriate clades. GenBank and strain ID associated with DNA sequences are shown on the left and right of the species name respectively.

Fig 4 presents a phylogenetic tree based on the near-full-length SSU rDNA sequences from a multiple alignment of 1636 total characters under the GTR+I+G model [-inL = 8287.9473; AIC = 16595.8945; freq A = 0.2517; freq C = 0.2001; freq G = 0.2626; freq T = 0.2855; R(A-C) = 0.898; R(A-G) = 3.775; R(A-T) = 2.795; R(C-G) = 0.6581; R(C-T) = 6.1285; R(G-T) = 1; Pinvar = 0.33; Shape = 0.6046]. Using *Caenorhabditis sinica* as an outgroup taxon, this tree inferred two monophyletic clades. One clade includes *Heterorhabditis* species and the other encompasses all remaining taxa. *P*. *huizhouensis* sp. nov. is in a highly supported monophyletic clade with other species of *Phasmarhabditis*, 2 isolates of *Agfa flexilis* and 3 isolates of *Angiostoma* spp. This clade is sister to *Rhabditella*. *P*. *huizhouensis* sp. nov. is different from *P*. *hermaphrodita*, *P*. *neopapillosa* and other unidentified *Phasmarhabditis* species. Fig 5 presents a phylogenetic tree based on the D2-D3 LSU rDNA sequences from a multiple alignment of 940 total characters under the GTR+I+G model [-inL = 6848.9263; AIC = 13717.8525; freqA = 0.237; freqC = 0.1867; freqG = 0.3007; freqT = 0.2757; R(A-C) = 0.463; R(A-G) = 2.4133; R(A-T) = 1.6894; R(C-G) = 0.5068; R(C-T) = 4.3753; R(G-T) = 1; Pinvar = 0.3433; Shape = 1.0758]. Using *C*. *sinica* as an outgroup taxon, *P*. *huizhouensis* sp. nov. is in a highly supported monophyletic clade with many isolates of *Phasmarhabditis*, 2 isolates of *Agfa* and 4 isolates of *Angiostoma*. This clade is sister to *Pellioditis*. The phylogenetic inferences based on two rDNA genes are largely consistent with each other. Both phylogenies reveal that *P*. *huizhouensis* sp. nov. is grouped with other nominal *Phasmarhabditis* species but in a unique branch.

## Discussion

The interrelationship between the genera *Phasmarhabditis* and *Pellioditis* remains unresolved, but in accord with both revisions by Andrássy (1983) and Sudhaus (2011) our study supported that the ‘*Papillosa*’ group of four known species comprising *P*. *papillosa*, *P*. *hermaphrodita*, *P*. *neopapillosa* and *P*. *tawfiki*, together with the new member of *P*. *huizhouensis* sp. nov. constitute a sound taxonomic group, based on an amalgamation of morphological and molecular data. The group is characterized by relatively short and wide buccal capsule, longitudinally striated lateral field with 3 or more ridges, relatively short female/hermaphroditic tail with prominent phasmids projecting from body contour, and male open peloderan bursa with 10 pairs of bursal papillae. However, the systematic relationships between these *Phasmarhabditis* taxa and their relatives in the subfamily Peloderinae are poorly understood, for example, two species of this subfamily *Pellioditis marina* and *P*. *mediterranea sensu* Andrássy [5] included by both phylogenetic trees (Figs 4 and 5) have been recently proposed fortransfer into another genus (*Litoditis* Sudhaus, 2011) [3]. This volatility of nematode systematics is a result of the scarcity of informative morphological/anatomical characters and a lack of DNA barcode sequences. The taxonomy of the whole Peloderinae group needs more work. Within the genus *Phasmarhabditis*, *P*. *huizhouensis* sp. nov. can be easily distinguished from other species except for *P*. *papillosa* by the female/hermaphroditic tail shape. *P*. *huizhouensis* sp. nov. is a morphologically cryptic species of *P*. *papillosa*, lacking typical autapomorphies except in males. The basis on which the former can be convincingly differentiated from *P*. *papillosa* are solely male caudal characters derived from available drawings for *P*. *papillosa*. Because of the previous inadequate morphological study and absence of DNA sequences for *P*. *papillosa*, further comparison at morphological as well as molecular levels between *P*. *huizhouensis* sp. nov. and *P*. *papillosa* is necessary when *P*. *papillosa* is recollected and sequenced. The excretory pore position of *P*. *huizhouensis* sp. nov. is significantly variable between females and a male observed in this study, which suggests the variability of excretory pore position for this species. However, a possibility of misidentification is unavoidable given the microscopic observation on the robust female adults in low magnifications. Thus, the excretory pore position of this species needs re-examination in the future.

Scanning electron micrographs of *Phasmarhabditis hermaphrodita* isolate ITD272 [8] and *P*. *huizhouensis* sp. nov. (Fig 3) reflect a number of apomorphic features shared by both species, e.g. the lip region characters (a triangular mouth opening formed by three pairs of lips with 6 labial and 4 cephalic sensilla, as well as small amphids) and lateral field morphology (longitudinally striated with 3 obvious middle-ridges). Female/hermaphroditic tail shapes are clearly distinct in the species but exhibit some degree of homology, i.e., a short conoid gradually tapered to a pointed terminus with prominent phasmids (see Fig 1F [8]) vs. a short cupola sharply to a pointed tip with ‘rod-like’ phasmids for *P*. *huizhouensis* sp. nov. (Fig 3I). Morphological features combined with molecular evidence are sufficiently for supposing a close systematic affinity in generic level between *P*. *huizhouensis* sp. nov. and *P*. *hermaphrodita*. However, other relatives of *P*. *huizhouensis* sp. nov. inferred from phylogenetic trees, such as *Angiostoma* and *Agfa* nematodes, are distantly related to the new species taxonomically. The genera belong to the distinct families Angiostomatidae and Agfidae respectively [35], and are clearly different from *P*. *huizhouensis* sp. nov. morphologically. *P*. *huizhouensis* sp. nov. differs from *Angiostoma* species in the type of stoma and esophagus (the former has a typically rhabditiform pharynx and a wide buccal capsule; the buccal capsule of *Angiostoma* spp. is wide or narrow which is conjoined by a pharynx with indistinct isthmus and a small-valvate or valveless basal bulb), cephalic sensory traits (6 labial as well as 4 cephalic sensory papillae vs. 6+6 papillae at the cephalic end), lateral field morphology (marked with longitudinal ridges vs. presence of lateral alae or lacking), and male caudal configuration (enveloped by an open peloderan bursa vs. an open leptoderan bursa or differing in the patterning of bursal papillae) [36–38]. *P*. *huizhouensis* sp. nov. is easily differentiated from *Agfa flexilis* by the morphology of the pharynx (a typically rhabditiform that is valvulated in the basal bulb for *P*. *huizhouensis* sp. nov. vs. a much longer one without valvular apparatus for *A*. *flexilis*) [39]. Nevertheless, molecular phylogenetic inference based on SSU rDNA sequences reveals that *Phasmarhabditis* isolates, *Angiostoma* species. and *A*. *flexilis* constitute a well-supported monophyletic clade (Fig 4), which conforms to a previous phylogenetic study among these genera [40]. Phylogenetic analysis based on the D2-D3 domain of LSU rDNA sequences in this study confirmed the close molecular relationship between these taxa (Fig 5). The reason is partly owing to the number of MOTUs (Molecular Operational Taxonomic Unit) which is insufficient to infer phylogenetic relationship between these genera, but the results reflect the inconsistencies between molecular phylogeny and traditional morphological classification. Revision of the systematics of the order Rhabditida based on previous morphological studies is needed.

*Phasmarhabditis huizhouensis* sp. nov. was isolated from rotting plant tissues, but is phylogenetically related to the slug-parasite *P*. *hermaphrodita* and other parasitic nematodes of *Angiostoma* spp. and *Agfa* species (Figs 4 and 5), suggesting that *P*. *huizhouensis* sp. nov. may be a facultative parasite. The ecology and host associations of *P*. *huizhouensis* sp. nov. remain unknown. It would be worthwhile to explore the potential of this *Phasmarhabditis* nematode to parasitize slugs as a local bio-control agent. Mass production of its dauer larvae would be essential if this species is proved to be capable of controlling slugs. In this study, the *C*. *elegans* bacterial food of *E*. *coli* OP50 was successfully used for culture of *P*. *huizhouensis* sp. nov., but no dauer stage was observed, suggesting that this bacterium may not be the best kind of food for this phasmarhabditid nematode, given that the growth rate of *Phasmarhabditis* species and its reproductive capacity for dauer larvae are strongly influenced by bacterial species [41]. The most suitable type of bacterial food for dauer larval formation and for mass production of *P*. *huizhouensis* sp. nov. require further study.
